# The causal relationship between atopic dermatitis and retinal detachment: A 2-sample Mendelian randomization analysis

**DOI:** 10.1097/MD.0000000000045813

**Published:** 2025-11-14

**Authors:** Xiazhen Liu, Hong Li, Taoran Tang, Yaqi Zhu, Shilin Zhu

**Affiliations:** a Nursing Department, The Second Affiliated Hospital of Hunan University of Chinese Medicine, Changsha, Hunan Province, China; b School of Nursing, Southern Medical University, Guangzhou, Guangdong Province, China.

**Keywords:** atopic dermatitis, causal inference, Mendelian randomization, retinal detachment

## Abstract

A 2-sample Mendelian randomization (MR) analysis was employed to determine the causal relationship between atopic dermatitis (AD) and retinal detachment (RD). Data related to AD and RD were retrieved from genome-wide association study databases, from which independent single nucleotide polymorphisms were selected as instrumental variables. MR analyses were performed using methods including inverse variance weighting, MR-Egger, and weighted median. Heterogeneity and pleiotropy were detected via Cochran’s *Q* test and MR-Egger intercept. Outliers were analyzed using Mendelian Randomization Pleiotropy RESidual Sum and Outlier (MR-PRESSO) and leave-one-out analysis. Reverse MR analysis was applied to determine the direction of the causal association between AD and RD. Inverse variance weighting analysis indicated a potential causal relationship between AD and RD (OR: 1.096; 95% confidence interval [CI]: 1.000–1.201; *P =* .048), and the weighted median approach also supported this conclusion (OR: 1.165; 95% CI: 1.023–1.328; *P =* .021). Additionally, reverse MR analysis did not indicate a causal relationship of RD to AD (OR: 0.993; 95% CI: 0.960–1.027; *P =* .704). No heterogeneity or horizontal pleiotropy was observed, suggesting robust results. This study confirmed a potential causal effect of AD on RD, and reverse MR analysis revealed that this causal association is unidirectional. Our results indicate a potential causal effect between AD and RD. It is recommended that healthcare providers be aware of the potential ocular complications in AD patients to ensure early detection and intervention, preventing irreversible progression.

## 1. Introduction

Atopic dermatitis (AD) is a common chronic inflammatory skin disease characterized by eczematous skin lesions, pruritus, and recurrent infections.^[1]^ It is one of the leading causes of the global burden of skin diseases.^[2]^ Retinal detachment (RD) is primarily manifested by visual disturbances, such as photopsia, shadows, visual field defects, and blurred vision.^[3]^ Recent large-scale epidemiological reports have indicated that RD, as one of the most common ophthalmic complications in patients with AD, has a significantly higher incidence rate than in the general population.^[4]^ A retrospective case study from Japan showed that the incidence of RD in AD patients ranges from 4% to 8%.^[5]^ Although some studies have suggested an association between AD and RD,^[5,6]^ this association was not observed in another study.^[7]^ The controversy among results and potential limitations (e.g., confounding factor interference and reverse causality) have rendered the causal relationship between AD and RD unclear.

Mendelian randomization (MR) is a statistical method that infers potential causal relationships between exposures and outcomes by utilizing genetic variants as instrumental variables (IVs) for exposures. This approach can minimize the interference of confounding factors and avoid reverse causality.^[8]^ In the present study, large-scale genome-wide association study (GWAS) datasets were used to determine the causal relationship between AD and RD via a 2-sample MR approach.

## 2. Materials and methods

### 2.1. Study design

To investigate the causal relationship between AD and RD, we retrieved data related to AD and RD from publicly available GWAS databases, selected eligible single nucleotide polymorphisms (SNPs), and employed a range of statistical methods to determine the causal association between AD and the risk of RD.

### 2.2. Data sources

The AD data were obtained from the integrative epidemiology unit open GWAS database, available at: https://gwas.mrcieu.ac.uk/datasets/ebi-a-GCST90027161/. This dataset includes a total of 7,96,661 samples, with 22,474 AD patients and 7,74,187 control cases, all from Europe. The number of SNPs included is 1,62,13,213. The RD data were sourced from the integrative epidemiology unit open GWAS database, available at: https://gwas.mrcieu.ac.uk/datasets/ebi-a-GCST90018911/. This database consists of 4,77,031 samples, including 6591 RD patients and 4,70,440 control cases, all from Europe. The number of SNPs included is 2,41,87,305.Comprehensive GWAS metadata are cataloged in Table 1.

**Table 1:** Summary of the GWAS included in the study.

	Database	Sample sizes	Number of cases	Number of controls	Number of SNPs	Population	References
AD	UK Biobank	7,96,661	22,474	7,74,187	1,61,21,213	European	PMID: 34454985
RD	UK Biobank	4,77,031	6591	4,70,440	2,41,87,305	European	PMID: 34594039

AD = atopic dermatitis, GWAS = genome-wide association study, PMID = PubMed identifier, RD = retinal detachment, SNP = single nucleotide polymorphism.

### 2.3. Selection of instrument variables

To obtain robust results in MR analysis, the following 3 assumptions must be met: relevance assumption: the selected genetic variants (usually SNPs) must be significantly associated with the exposure factor; independence assumption: the genetic variants must be independent of potential confounders; exclusivity assumption: the genetic instruments must influence the outcome solely through the exposure factor and cannot directly affect the outcome through other pathways.^[9]^ Based on these assumptions, we selected SNPs closely associated with AD (*P* < 5 × 10^−8^) as IVs. Additionally, to ensure that the r² value is <0.001 and the genetic distance is >10,000 kb, while maintaining the independence of SNPs, we excluded SNPs in linkage disequilibrium. Using Mendelian Randomization Pleiotropy RESidual Sum and Outlier (MR-PRESSO) tests, we identified potential outliers in the genetic instruments and ultimately obtained IVs significantly associated with AD.

### 2.4. Statistical processing

All statistical analyses in this study were conducted using R 4.4.1 software, the TwoSampleMR package, and the MR-PRESSO package. Causal effects between AD and RD were estimated using inverse variance weighting (IVW), MR-Egger, weighted median (WM), simple model, and weighted model, with IVW as the primary method. Additionally, Cochran’s *Q* statistic was used to test for heterogeneity among the IVs, with *P* > .05 indicating no heterogeneity.^[10]^ The MR-Egger regression intercept was used to test for potential horizontal pleiotropy, with *P* > .05 suggesting no horizontal pleiotropy.^[11]^ MR-PRESSO tests were employed to detect outliers and horizontal pleiotropy. Finally, a leave-one-out sensitivity test was performed to assess the impact of individual SNPs on the causal association estimate, further validating the robustness of the study’s results. We also used the reverse MR analysis to determine the direction of the causal relationship between exposure and outcome. Specifically, reverse MR estimates the causal effect of the outcome on the exposure by using IVs associated with the outcome. When both the original MR and reverse MR analyses yield significant causal associations for a pair of traits, the causal relationship between the pair of traits is considered bidirectional rather than unidirectional.

## 3. Results

### 3.1. Causal relationship of AD on RD

**Figure 1.**
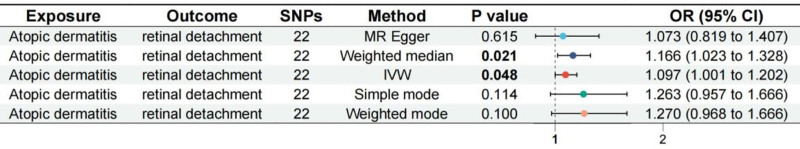
Forest plot of the causal relationship between AD and RD. CI = confidence interval, AD = atopic dermatitis, IVW = inverse variance weighting, MR = Mendelian randomization, OR = odds ratio, RD = retinal detachment, SNP = single nucleotide polymorphism.

**Figure 2.**
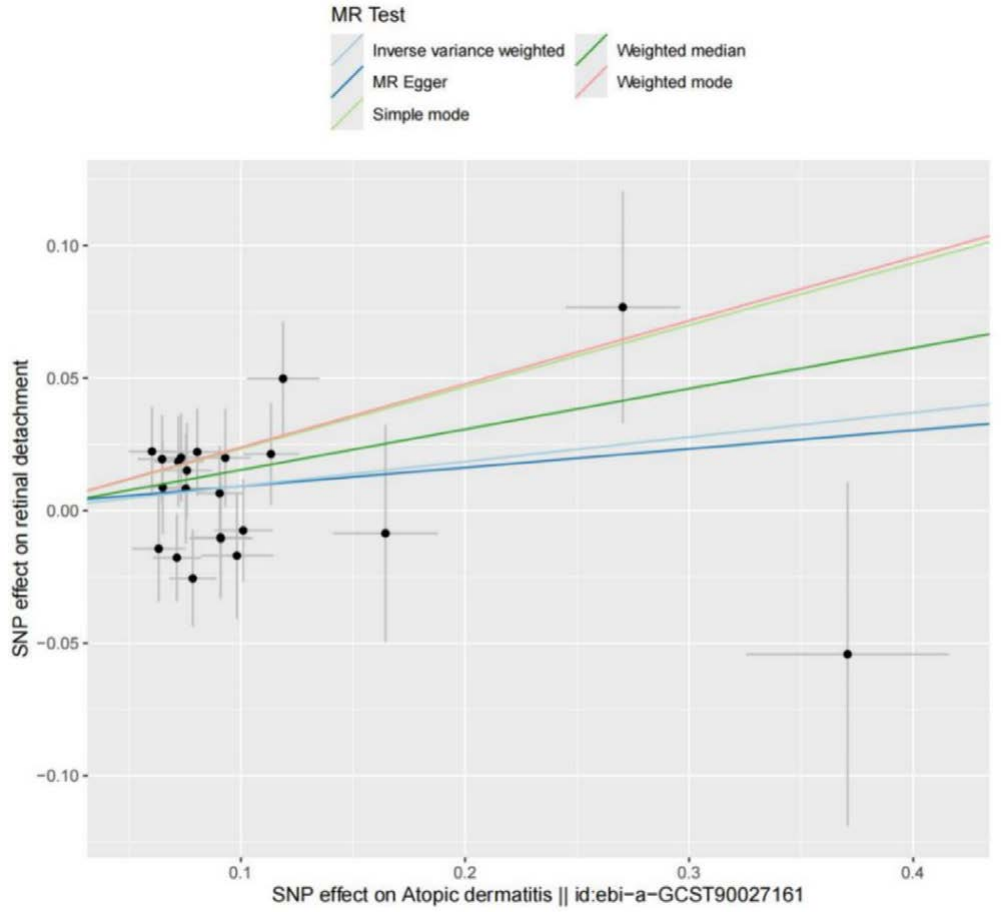
Scatter plot of the causal relationship between AD and RD. AD = atopic dermatitis, MR = Mendelian randomization, RD = retinal detachment.

We selected 22 SNPs from GWAS data as IVs. All F-statistics for these SNPs exceeded 10, indicating the absence of weak instrument bias and confirming the reliability of the analyses. Causal effect estimates from 2 methods – IVW (odds ratio [OR]: 1.096; 95% confidence interval [CI]: 1.000–1.201; *P* = .048) and WM (OR: 1.165; 95% CI: 1.023–1.328; *P* = .021) – were statistically significant (*P* < .05). Collectively, these results suggest that AD is associated with increased genetic susceptibility to RD. Notably, while both OR values reached statistical significance, they were numerically close to 1.0, indicating that the overall magnitude of AD’s causal effect on RD is small. Thus, although the current causal estimates confirm an “association,” whether they can directly guide clinical decision-making requires further investigation. A forest plot illustrating the causal relationship between AD and RD is presented in Figure 1, and the corresponding scatter plot is shown in Figure 2.

### 3.2. Sensitivity analysis

**Figure 3.**
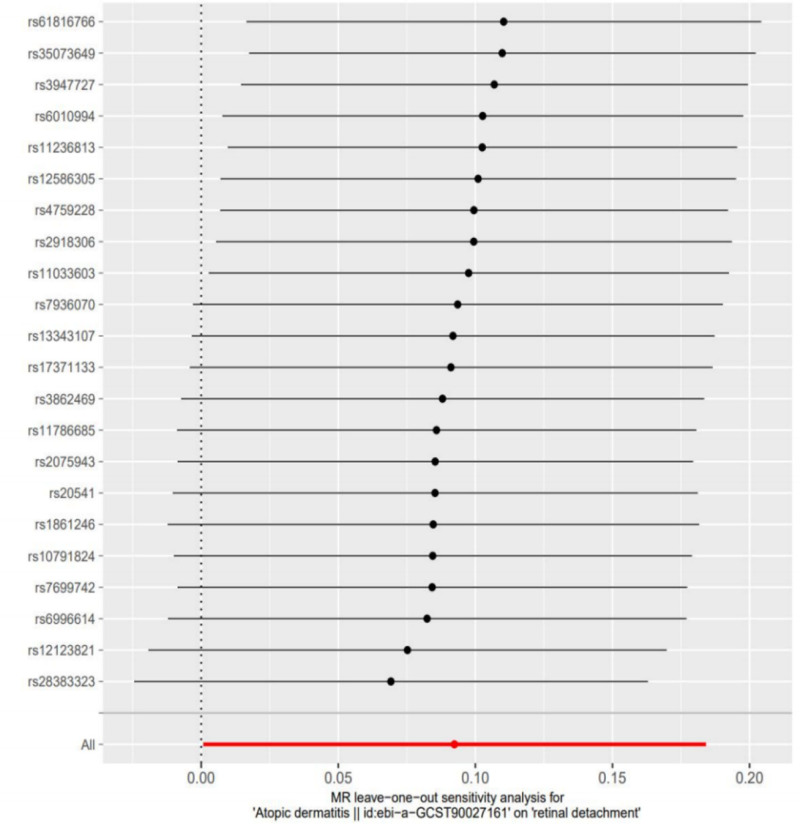
Leave-one-out sensitivity analysis of the causal relationship between AD and RD. AD = atopic dermatitis, MR = Mendelian randomization, RD = retinal detachment.

To ensure the robustness of the results, we performed sensitivity analysis. Cochran’s *Q* test showed *P* = .42, indicating no heterogeneity. The MR-Egger regression intercept (*P* = .86) did not show horizontal pleiotropy. Finally, we conducted a leave-one-out sensitivity analysis, and the results showed that even when SNPs were removed 1 by 1, the estimated results did not show significant differences, further confirming the robustness of the final results (Fig. 3).

### 3.3. Reverse MR analysis results

**Figure 4.**
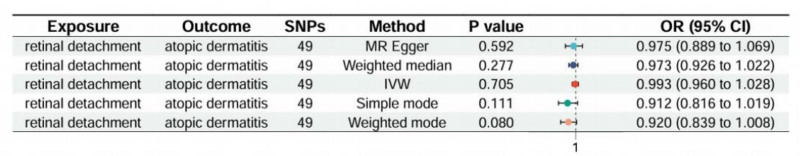
Forest plot of the causal relationship between RD and AD. CI = confidence interval, AD = atopic dermatitis, IVW = inverse variance weighting, MR = Mendelian randomization, OR = odds ratio, RD = retinal detachment, SNP = single nucleotide polymorphism.

**Figure 5.**
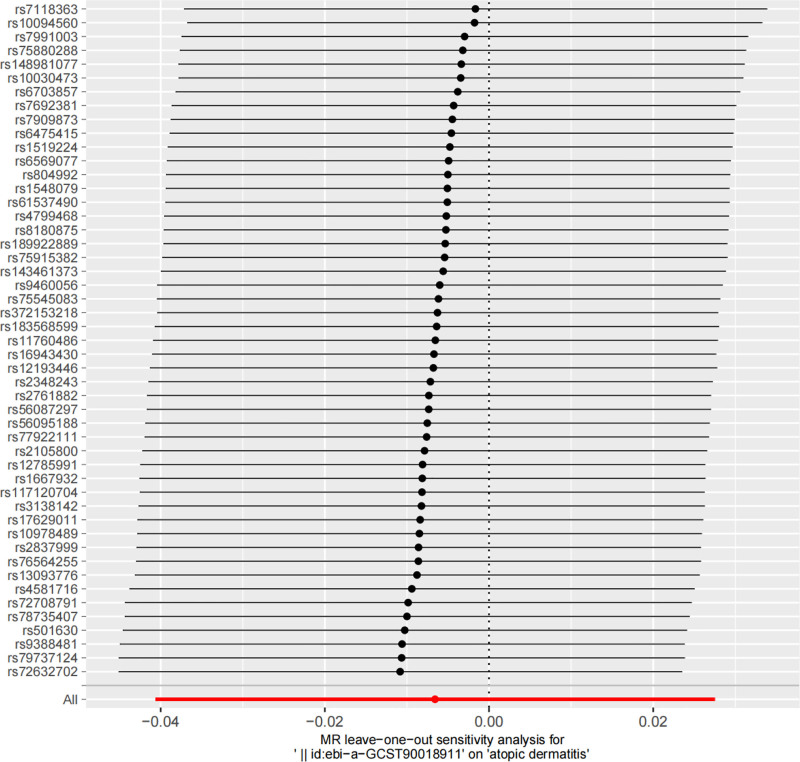
Leave-one-out sensitivity analysis of the causal relationship between RD and AD. AD = atopic dermatitis, MR = Mendelian randomization, RD = retinal detachment.

As shown in Figure 4, the IVW (OR: 0.993; 95% CI: 0.960–1.028; *P* = .705) did not show a significant causal relationship between RD and AD. Cochran’s *Q* test (*P* = .83) and the MR-Egger regression intercept (*P* = .66) indicated no heterogeneity or horizontal pleiotropy. Additionally, the leave-one-out sensitivity analysis confirmed the robustness of this result (Fig. 5).

## 4. Discussion

AD is a common skin disorder with a lifetime prevalence of up to 20%. It exhibits broad clinical manifestations, affecting over 90% of the body’s skin surface in severe cases,^[12]^ imposing a substantial burden on patients and their families. RD is a sight-threatening ocular condition. Its incidence has risen steadily, increasing from 15.6 cases per 1,00,000 individuals in 2005 to 24.8 cases per 1,00,000 in 2021.^[13]^ Given these trends, greater emphasis is urgently needed on the prevention and management of both AD and RD.

In 1937, Balyeat first reported that RD could occur secondarily to AD.^[14]^ Since then, a growing body of research has highlighted a potential association between the 2 conditions. A study by Kishan et al^[15]^ demonstrated that the incidence of RD in AD patients was 3.22 times higher than that in the general population. In an observational study of 6 AD patients conducted by Jothi et al,^[16]^ 8 out of 12 eyes were found to have RD. Additionally, another study noted that AD patients typically develop RD during the second and third decades of life.^[17]^

The specific mechanism by which AD elevates the risk of RD remains unclear; however, previous studies have proposed several potential explanations. The first is mechanical trauma. Oka et al^[18]^ compared the clinical characteristics of RD secondary to trauma versus RD associated with AD. They found that both traumatic RD and AD-related RD shared similar fundus manifestations: tractional retinal tears at the border of the vitreous base. Additionally, retinal tears in AD patients typically affect the temporal region of the retina – a finding that further suggests these tears result from ipsilateral hand scratching.^[19]^ This conclusion was supported and validated in studies by Jothi et al^[16]^ and Lee et al,^[20]^ as AD patients, due to their characteristic pruritus (itching), develop a habitual tendency to rub or tap their eyes. Such actions cause mechanical ocular trauma, thereby increasing the risk of RD.

A second proposed mechanism is inflammation. Since both the skin and retina originate from the neuroectoderm, AD not only triggers cutaneous inflammation but may also induce intraocular inflammation that contributes to RD. Accordingly, studies have suggested that RD in AD patients arises from chronic inflammation involving the vitreous base, peripheral retina, and ciliary body.^[21,22]^ Additionally, Mikio et al^[5]^ noted in their research that the most common characteristic of AD associated with RD is bilateral involvement – a feature that may be linked to severe facial AD inflammation.

Additionally, research suggests this association may be linked to AD treatment. The globally recognized first-line therapy for AD is topical corticosteroids, which are known to elevate intraocular pressure and contribute to the development of ocular disorders.^[15]^ A retrospective study from Japan^[5]^ demonstrated that the introduction of nonsteroidal treatments for AD – such as tacrolimus and cyclosporine – was associated with a significant reduction in AD associated with RD. This finding further supports the notion that RD secondary to AD may be related to its treatment.

The present study identified a statistically significant causal association between AD and genetic susceptibility to RD, as evidenced by results from 2 analytical methods: IVW (OR: 1.096; 95% CI: 1.000–1.201; *P =* .048) and WM (OR: 1.165; 95% CI: 1.023–1.328; *P =* .021). However, when interpreting the OR values – both of which are close to 1.0 – it is important to note that the magnitude of the causal effect between AD and RD is small. Consequently, the clinical utility of these findings (e.g., for risk screening or the development of intervention strategies) should be evaluated objectively.

From a clinical standpoint, the current findings do not meet the clinical threshold for an “effect size with clear actionable value”(OR > 1.2). This means AD is not currently supported as a core screening indicator for RD – patients cannot be classified as “high-risk for RD” nor targeted interventions initiated solely based on their AD medical history or genetic background. Simultaneously, therapies targeting AD (e.g., anti-inflammatory or immunomodulatory agents) are unlikely to drive a meaningful reduction in RD risk by mitigating AD severity, so AD should not be regarded as a priority intervention focus for preventing RD.

Nevertheless, this association retains relevance for certain clinical contexts: For patients with a confirmed diagnosis of AD, clinicians may incorporate enhanced ocular health monitoring into routine care – such as regular fundus examinations – and include RD in the “list of potential complications to monitor” for AD, enabling early detection and management. Additionally, future research could focus on high-risk subgroups of AD patients with comorbid ocular conditions; if such subgroups exhibit a significantly elevated odds ratio (OR > 1.2, for example) between AD and RD, this may provide a basis for targeted risk management.

While the present study confirmed a potential causal effect between AD and RD using MR analysis, it is not without limitations. For instance, our research relied solely on GWAS data from European populations, which may restrict the generalizability of the findings to other ethnic groups. In future work, integrating cross-ethnic GWAS datasets could clarify differences in the causal association between AD and RD across populations, thereby enhancing the generalizability of the results. Additionally, our study generated only statistical outcomes and did not delve into the mechanisms underlying how AD contributes to RD – an area that warrants further investigation in subsequent research.

## 5. Conclusions

In this study, we used the 2-sample MR method to infer the causal relationship between AD and RD, revealing the potential impact of AD on the progression of RD. This not only expands our understanding of the pathogenesis of RD but also provides new insights for clinical diagnosis and treatment. Therefore, we recommend that all healthcare professionals involved in the diagnosis, treatment, and care of AD patients be aware of the potential ocular complications in these patients to ensure early detection and intervention, preventing irreversible progression.

## Author contributions

**Formal analysis:** Xiazhen Liu.

**Funding acquisition:** Shilin Zhu.

**Methodology:** Yaqi Zhu.

**Supervision:** Hong Li, Taoran Tang, Shilin Zhu.

**Validation:** Hong Li, Taoran Tang, Yaqi Zhu.

**Writing – original draft:** Xiazhen Liu.

**Writing – review & editing:** Xiazhen Liu, Shilin Zhu.
